# CTAG-Containing Cleavage Site Profiling to Delineate *Salmonella* into Natural Clusters

**DOI:** 10.1371/journal.pone.0103388

**Published:** 2014-08-19

**Authors:** Le Tang, Wei-Qiao Liu, Xin Fang, Qiang Sun, Song-Ling Zhu, Chun-Xiao Wang, Xiao-Yu Wang, Yong-Guo Li, Da-Ling Zhu, Kenneth E. Sanderson, Randal N. Johnston, Gui-Rong Liu, Shu-Lin Liu

**Affiliations:** 1 Genomics Research Center (one of The State-Province Key Laboratories of Biomedicine-Pharmaceutics of China), Harbin Medical University, Harbin, China; 2 Department of Biopharmaceutical Sciences, Harbin Medical University, Harbin, China; 3 HMU-UCFM Centre for Infection and Genomics, Harbin Medical University, Harbin, China; 4 Department of Microbiology and Infectious Diseases, University of Calgary, Calgary, Canada; 5 Department of Infectious Diseases of First Affiliated Hospital, Harbin Medical University, Harbin, China; 6 College of Pharmacy, Daqing Campus, Harbin Medical University, Daqing, China; 7 Department of Biochemistry and Molecular Biology, University of Calgary, Calgary, Canada; University of Exeter Medical School, United Kingdom

## Abstract

**Background:**

The bacterial genus *Salmonella* contains thousands of serotypes that infect humans or other hosts, causing mild gastroenteritis to potentially fatal systemic infections in humans. Pathogenically distinct *Salmonella* serotypes have been classified as individual species or as serological variants of merely one or two species, causing considerable confusion in both research and clinical settings. This situation reflects a long unanswered question regarding whether the *Salmonella* serotypes exist as discrete genetic clusters (natural species) of organisms or as phenotypic (e.g. pathogenic) variants of a single (or two) natural species with a continuous spectrum of genetic divergence among them. Our recent work, based on genomic sequence divergence analysis, has demonstrated that genetic boundaries exist among *Salmonella* serotypes, circumscribing them into clear-cut genetic clusters of bacteria.

**Methodologies/Principal Findings:**

To further test the genetic boundary concept for delineating *Salmonella* into clearly defined natural lineages (e.g., species), we sampled a small subset of conserved genomic DNA sequences, i.e., the endonuclease cleavage sites that contain the highly conserved CTAG sequence such as TCTAGA for XbaI. We found that the CTAG-containing cleavage sequence profiles could be used to resolve the genetic boundaries as reliably and efficiently as whole genome sequence comparisons but with enormously reduced requirements for time and resources.

**Conclusions:**

Profiling of CTAG sequence subsets reflects genetic boundaries among *Salmonella* lineages and can delineate these bacteria into discrete natural clusters.

## Introduction

Since the first isolation of a *Salmonella* pathogen from a typhoid patient in 1881, more than 2500 different *Salmonella* types have been documented [1], [2]. Based on their differences in the somatic (O) and flagellar (H) antigens, the *Salmonella* bacteria are classified into serotypes by the Kauffmann-White scheme [3]. Initially, the *Salmonella* serotypes were treated as individual species each having a Latinized scientific name such as *Salmonella typhi* and *Salmonella typhimurium*, but in the 1980s all *Salmonella* serotypes were combined into one species (*Salmonella enterica* [4]) or two species (*Salmonella enterica* and *Salmonella bongori* [5]) as serological variants (serovars [6]) due largely to the extraordinarily high genetic similarity among them, which has caused confusion in research and clinical settings. Indeed, all *Salmonella* serotypes have very similar genetic backgrounds as revealed by DNA-DNA re-association [7], comparison of genome structures [8], [9] and genomic sequencing [10]–[12], but on the other hand they may differ radically in pathogenic properties. For example, whereas many *Salmonella* serotypes may cause self-limited gastroenteritis (such as *S. typhimurium*, *S. enteritidis*, etc.) or may be virtually non-pathogenic to humans, a few may elicit potentially fatal systemic infections, such as *S. typhi* that causes typhoid [13]. The dynamic and confusing *Salmonella* taxonomy reflects long lasting uncertainties about the phylogenetic status of *Salmonella*: do they dwell in nature as discrete genetic clusters of organisms or as phenotypic variants of a single (or two) natural species with a continuous spectrum of genetic divergence among them?

To examine this issue, we have tested two hypotheses: first, that all *Salmonella* serotypes form a common gene pool in which DNA exchange occurs readily so that each member has an equal chance to become a different pathogen (e.g., infecting a different host species or causing a different disease) by acquiring appropriate genetic material and incorporating it into the genome; and second, that each *Salmonella* type (e.g. a serological or pathogenic type) is already an established biological unit, members of which have a common and highly stable genome structure as a result of natural selection over long evolutionary time.

If the first hypothesis is correct, all *Salmonella* serotypes should be combined into just one species. If the second hypothesis is correct, each *Salmonella* type is a genetically well-defined natural species. The first hypothesis would be supported by demonstration of a continuous spectrum of genetic divergence among different *Salmonella* types and, conversely, the second hypothesis would be validated by demonstration of clear-cut genetic boundaries among different *Salmonella* types as a result of genetic isolation and independent accumulation of mutations over long evolutionary time. Findings that support either hypothesis will lead to novel insights into the population structure of *Salmonella* and the mechanisms of divergence that have occurred during their adaptation to different environments (e.g., a particular host) during their evolution. A key step towards an answer is to elucidate whether the individual *Salmonella* types can be grouped into discrete, well separated genetic clusters. The classical method for *Salmonella* differentiation is serological typing, but a serotype may be polyphyletic. For example, the antigenic formula of 6,7∶c∶1,5 is common to multiple distinct pathogens, e.g., *S. paratyphi* C, *S. choleraesuis* and *S. typhisuis*, which infect different hosts or cause different diseases. Furthermore, based on serotyping only, one cannot judge whether the *Salmonella* serotypes are genetically well isolated from one another or whether some might be genetic “intermediates” between other serotypes.

Recently, we provided evidence showing that *Salmonella* exist in discrete genetic clusters isolated by clear-cut genetic boundaries [14]. However, that work was based on whole genome analysis. To further test the robustness of the genetic boundary concept in delineating *Salmonella* into clearly defined natural lineages (e.g., species), we sampled a small subset of conserved genomic DNA sequences, i.e., the endonuclease cleavage sites that contain the CTAG sequence such as TCTAGA for XbaI. As enteric bacteria tend to eliminate the short sequence CTAG by the Very Short Patch (VSP) repair mechanism [15], endonuclease cleavage sites containing CTAG are scarce and highly conserved in *Salmonella*. We found that profiling of the CTAG-containing cleavage sequences could resolve the genetic boundaries as reliably and efficiently as whole genome analyses but with enormously reduced requirements for time and resources.

## Results

### Monophyletic *Salmonella* serotypes have highly conserved cleavage patterns by CTAG-containing endonucleases

**Figure 1:**
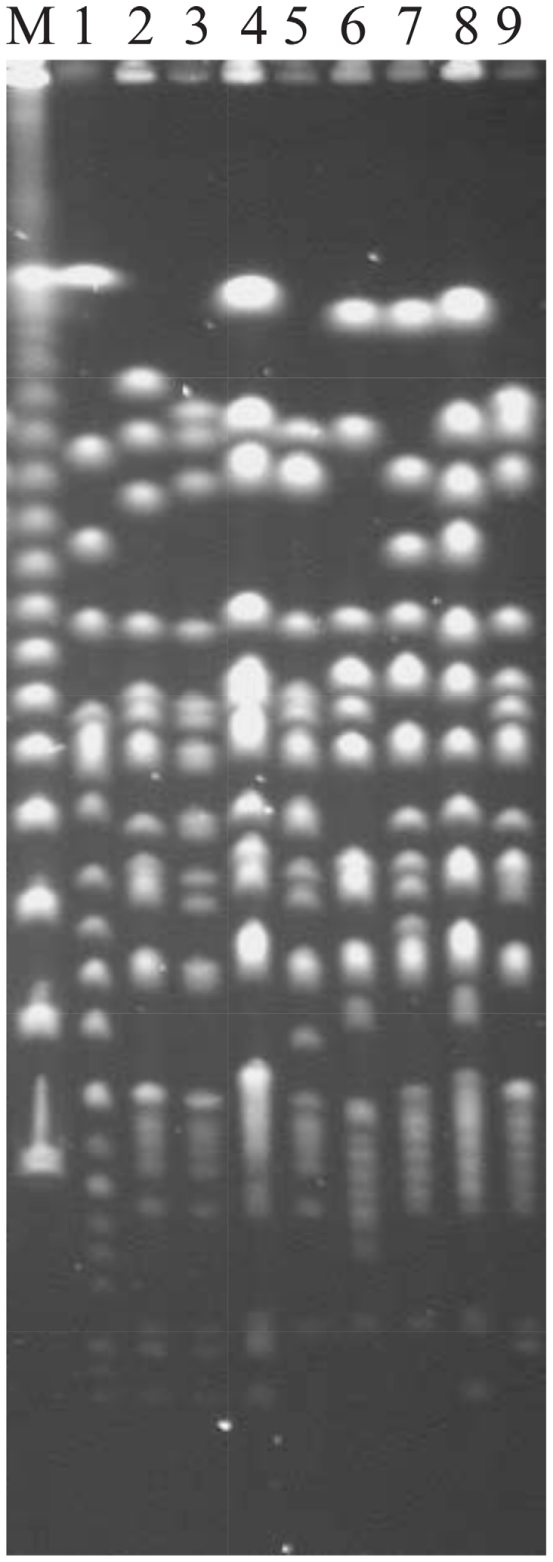
Diversity of cleavage patterns with AvrII among *S. gallinarum* wild type strains. Lanes: 1, molecular size marker (λDNA concatemer); 2, RKS5078; 3, SGSC2423; 4, SGSC2292; 5, SGSC2293; 6, R1481; 7, R1482; 8, R1483; 9, SARB21; 10, 287/91. *S. pullorum* RKS5078 (Lane 2) is included here for a comparison with the *S. gallinarum* strains.

**Figure 2:**
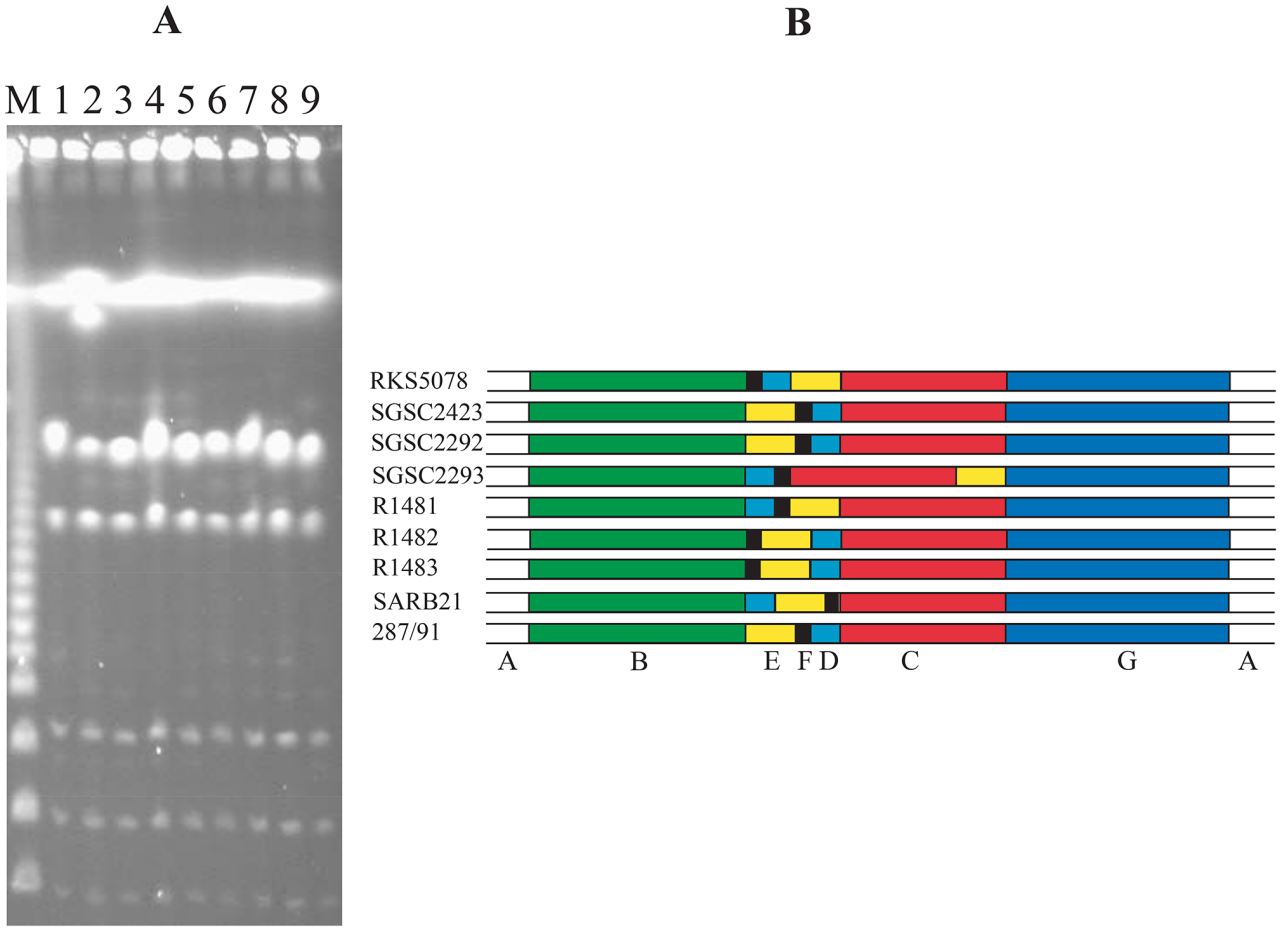
Genomic rearrangements of *S. gallinarum* strains. (A), PFGE patterns of incomplete I-CeuI cleavage cleaved genomic DNA. Lanes: same as in Figure 1; (B) Genome maps based on I-CeuI data in (A). As seen here, wild type strains have the seven I-CeuI fragments organized differently, with six genome types being resolved among the 8 strains of *S. gallinarum*. The map of *S. pullorum* RKS5078 is presented here for a comparison with the *S. gallinarum* strains.

It has been well documented that wild type strains of a monophyletic *Salmonella* serotype exhibit highly similar endonuclease cleavage patterns for XbaI and BlnI/AvrII on PFGE, such as *S. typhimurium* [16], *S. typhi* [17] or *S. paratyphi* A [18], in comparison with the diverse cleavage patterns seen in polyphyletic serotypes such as *S. paratyphi* B [19]. However, when we looked at *S. gallinarum*, known as a monophyletic *Salmonella* serotype, we saw considerable diversity of cleavage patterns among wild type strains for the endonucleases that have CTAG in the cleavage sites, as illustrated by AvrII cleavage in Figure 1. To determine whether the diversity of cleavage patterns was created by nucleotide base changes (leading to addition or deletion of cleavage sites) or by genomic rearrangements (changing the lengths of DNA fragments between the cleavage sites), we compared the genome structures of these strains. Analysis of incomplete I-CeuI cleavage products of the bacterial genomes showed that these strains had their genomes rearranged in several ways by recombination between *rrn* operons (Figure 2; for details about I-CeuI and *rrn*-mediated genomic rearrangements, see [8], [20]), suggesting that at least part of the diverse cleavage patterns have resulted from genomic rearrangements.

**Figure 3:**
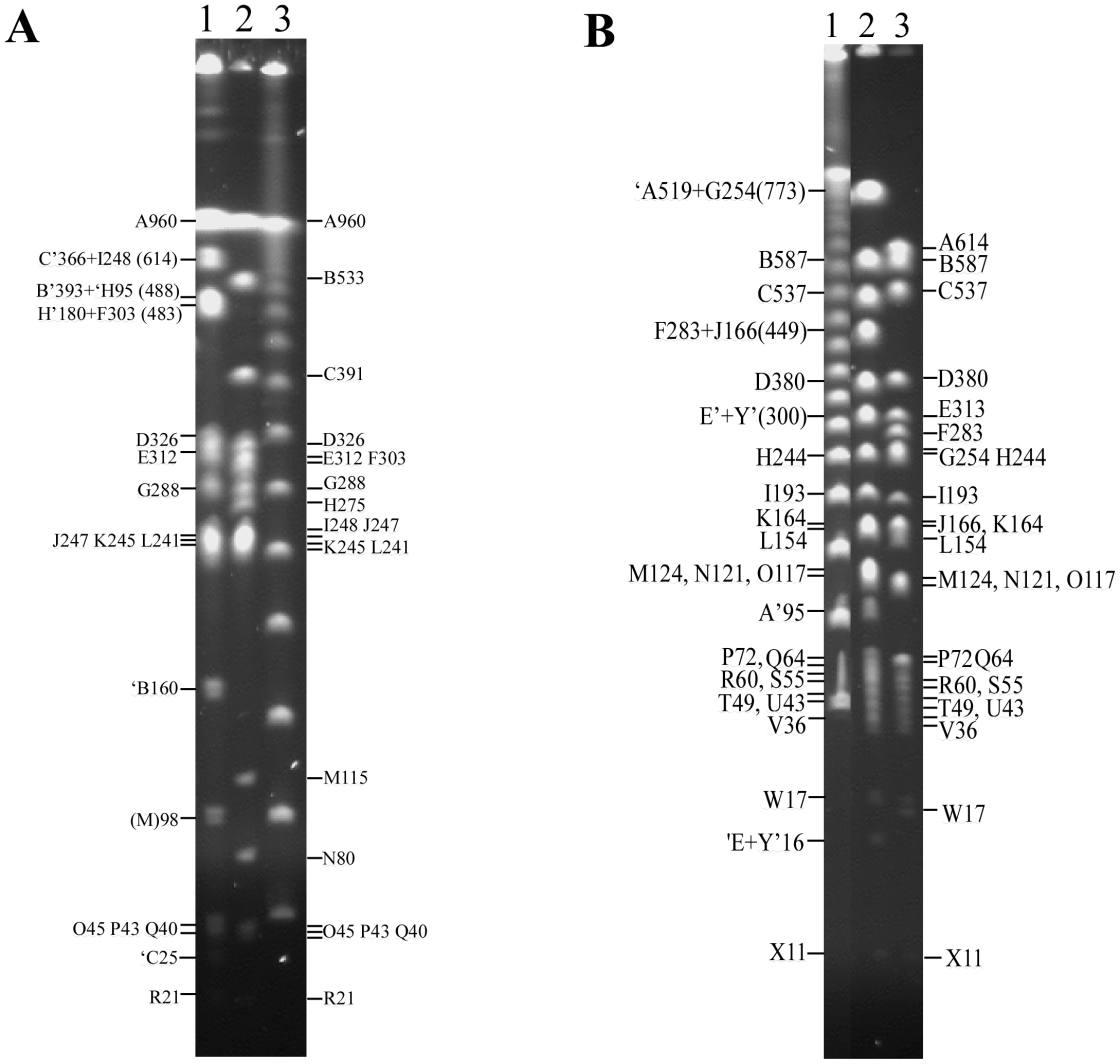
XbaI and AvrII cleavage patterns of *S. gallinarum* strains 287/91 and SARB21 after PFGE separation. (A) XbaI cleavage. Lanes: 1, SARB21; 2, 287/91; 3, λDNA as molecular size marker. (B) AvrII cleavage. Lanes: 1, λDNA as molecular size marker; 2, SARB21; 3, 287/91. Letter designations are for strain 287/91; the same letters are used for homologous fragments in strain SARB21. In the designation of fragments in SARB21, C′ means a fragment homologous to C in 287/91 but truncated on the right-hand part by genomic rearrangement, and ‘C means truncation on the left-hand part of the fragment.

**Figure 4:**
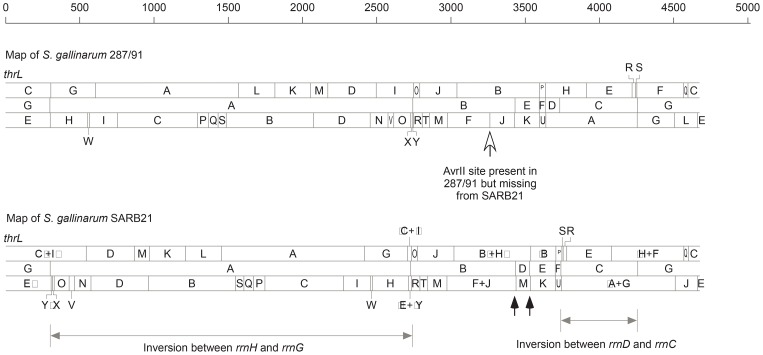
Physical map comparison between *S. gallinarum* strains 287/91 and SARB21. The map of SARB21 was reported previously [21]; here letter designations for the cleavage fragments of SARB21 have been changed according to the homologues in strain 287/91 for the convenience of comparison. Note that all XbaI, I-CeuI and AvrII (maps from top to bottom) cleavage sites are conserved in the two strains except the AvrII site between fragments F and J in 287/91 (open arrow), which is missing from SARB21. Lines with solid arrowheads at both ends indicate the ranges of genomic inversions via *rrn*-mediated recombination between the two strains and filled arrows indicate recombination sites that have resulted in the translocation of I-CeuI fragment D.

Next, we needed to determine whether the genomic rearrangements have just altered the lengths between pairs of AvrII sites or might have disrupted any of the AvrII cleavage sites (it is highly unlikely that genomic rearrangements may create new CTAG-containing cleavage sites). For this, we compared two representative *S. gallinarum* strains, SARB21 and 287/91 (Figure 3), which were previously mapped [21] or sequenced [22], respectively. We analyzed the genome maps of the two strains by matching the homologous cleavage sites between them for XbaI and AvrII, in addition to I-CeuI. We found that, as expected, most of the cleavage pattern differences between *S. gallinarum* SARB21 and 287/91 could be accounted for by two inversions (one between *rrnH* and *rrnG* and one between *rrnD* and *rrnC*) and one translocation (I-CeuI Fragment D), all of which massively altered the lengths of homologous genomic DNA segments flanked by the CTAG-containing endonuclease cleavage sites (Figure 4). The *rrnH*-*rrnG* inversion made XbaI Fragments C and I to join, forming Fragments C′+I′ and ‘C+’I (XbaI C391 and I248 missing and XbaI C′+I′ 614 and ‘C+’I 25 appearing in strain SARB21 relative to 287/91), along with corresponding changes in AvrII cleavage (See Figures 3 and 4). The I-CeuI Fragment D translocation and *rrnD*-*rrnC* inversion resulted in XbaI Fragment B533 splitting to B′ and ‘B, with B’ joining H′ to become B′+H′488, and a truncated ‘B160 fusing with ‘H+F to create a 483 kb segment, along with corresponding changes in AvrII cleavage (See Figures 3 and 4). The only unique AvrII cleavage site is present in strain 287/91 at about 3250 kb from gene *thrL* (Figure 4, indicated by the open arrowhead), probably as a result of a mutation in the corresponding AvrII cleavage site in strain SARB21 rather than creation of an AvrII cleavage site in strain 287/91.

### Conservation of CTAG-containing endonuclease cleavage sites within other representative *Salmonella* serotypes

To assess the extent of conservation of the CTAG-containing endonuclease cleavage sites, we conducted systematic comparisons of the cleavage locations on the genome for XbaI among strains of representative *Salmonella* serotypes, numbering the cleavage sites sequentially according to their locations on the genome of *S. typhimurium* LT2. Cleavage sites present in any strains but not in LT2 were not numbered. As exemplified by the six *S. typhimurium* strains, the XbaI cleavage sites were highly conserved within a *Salmonella* lineage, consistent with the findings by the PFGE techniques. LT2 has 27 XbaI cleavage sites numbered XbaI 1–27 (Table 1), most of which were conserved among all six compared *S. typhimurium* strains. Of particular significance, as many as over one third of the 27 XbaI cleavage sites fell in intergenic sequences, strongly suggesting the potential importance of these sequences. Among the six *S. typhimurium* strains, we found two kinds of differences in XbaI sites: presence/absence and presence/degeneracy. The non-conserved XbaI cleavage sites have largely resulted from recent insertions such as prophages or phage remnants (Supplementary Table S1). The sequence degeneracy of the XbaI cleavage sites can be illustrated by XbaI 9, which was present in LT2 but not in any of the other five *S. typhimurium* strains due to nucleotide substitution, changing the XbaI cleavage site TCTAGA to TCCAGA and leading to the replacement of leucine in LT2 by proline in the other five *S. typhimurium* strains.

**Table 1:** Comparison of XbaI cleavage sites among six *S. typhimurium* strains.

No.^a^	Gene ID^b^	Start^c^	End^d^	LT2	14028S	D23580	SL1344	ST474	UK1
						406086			
XbaI 1	STM0557	614804	616456	615218	615912	654028	614706	614706	615911
XbaI 2	STM1331–STM1332	1409765	1410086	1409947	1419923	1406459	1366800	1366800	1368113
XbaI 3	STM1377–STM1378	1459616	1459925	1459627	1469603	1456139	1416479	1416479	1417794
XbaI 4	STM1622–STM1623	1711483	1711701	1711569	1721546	1704084	1668422	1668422	1669738
XbaI 5	STM2093	2174120	2175112	2174259	2225811	2197914	2171974	2171974	2174004
XbaI 6	STM2292	2399433	2400215	2399521	2451074	2423187	2397237	2397237	2399266
XbaI 7	STM2394–STM2394	2505894	2506054	2506054	2557606	2529720	2503770	2503770	2505799
XbaI 8	STM2584–STM2585	2730470	2731355	2730547	2782100	2754927	2728264	2728264	2730311
XbaI 9	STM2616	2762064	2762627	2762167					
XbaI 10	STM2658	2799958	2800030	2800018	2818163	2790985	2800431	2800431	2802188
							2824797	2824797	
					2853935	2826674			
							2877794	2877794	
							2888767	2888767	
XbaI 11	STM2742	2880444	2881637	2880896	2901121	2873861	2903461	2903461	2849374
XbaI 12	STM2747	2887401	2888219	2887451	2907676	2880416	2910016	2910016	2855929
XbaI 13	STM2767	2908420	2910402	2908601	2928836	2901586	2931177	2931177	2877089
XbaI 14	STM2767	2908420	2910402	2909761	2929996	2902746	2932337	2932337	2878249
XbaI 15	STM3397	3570307	3570379	3570365	3584223	3593463	3591847	3591847	3531830
XbaI 16	STM3405–STM3406	3576805	3576935	3576883	3590741	3599981	3598365	3598365	3538348
XbaI 17	STM3443–STM3444	3597851	3597922	3597875	3611733	3620973	3619357	3619357	3559340
XbaI 18	STM3594–STM3595	3765983	3766163	3766042	3779743	3788912	3787367	3787368	3727350
XbaI 19	STM3646	3833687	3834661	3834463	3848154	3857340	3855778	3855779	3795761
XbaI 20	STM3714–STM3715	3910705	3910806	3910806	3924498	3933683	3932120	3932121	3872105
XbaI 21	STM3785	3984439	3985182	3984523	3998215	4007398	4005836	4005837	3945822
XbaI 22	STM3846–STM3847	4053276	4054076	4053903	4067596	4076779	4075214	4075215	4015203
XbaI 23	STM3890	4101759	4101831	4101769	4115462	4124645	4123076	4123077	4063068
XbaI 24	STM4178	4396303	4396375	4396313	4409893	4419063	4417645	4417646	4357499
XbaI 25	STM4285	4525350	4527497	4526230	4539772	4548929	4547522	4547523	4487378
XbaI 26	STM4362	4604955	4606235	4606231	4619062	4628219	4626812	4626813	4566668
XbaI 27	STM4524	4777606	4779015	4778149	4790982	4800127	4798732	4798733	4738586

**Notes:**

a , XbaI cleavage sites numbered according to their appearance order in LT2 starting from the “beginning” of the genome, i.e., gene thrL.

b , One gene ID, such as STM0557, means that the XbaI cleavage site falls in a gene; two gene IDs, such as STM1331–STM1332, mean that the XbaI cleavage site falls in an intergenic region between two genes.

c and d , start and end nucleotide positions, respectively of a gene or an intergenic region between two genes.

Within each of the other *Salmonella* serotypes analyzed, the CTAG-containing cleavage sites were also highly conserved, with the main differences among the wild type strains being additional cleavage sites in prophages or genomic islands (Supplementary Table S1). For example, *S. heidelberg* SL476 had three large genomic islands, 58, 30 and 42 kb in size, respectively, all containing multiple XbaI cleavage sites; the 42 kb island, present in *S. heidelberg* SL476 but not in *S. heidelberg* B182, contained as many as seven additional XbaI cleavage sites within a 20 kb region (Supplementary Table S1). Other endonucleases (e.g., SpeI) having CTAG in the cleavage sites had similar situations as XbaI (data not shown). The overall conservation of the CTAG-containing endonuclease cleavage sequences in the *Salmonella* genomes makes it possible to use these endonucleases for the identification of *Salmonella* isolates. For this, the distinctness of cleavage patterns of endonucleases with CTAG in the cleavage sequences across different *Salmonella* serotypes (or lineages; a monophyletic *Salmonella* serotype is equivalent to “a *Salmonella* lineage” but a polyphyletic *Salmonella* serotype contains two or more *Salmonella* lineages) would have to be documented.

### CTAG endonuclease cleavage patterns are distinct across *Salmonella* lineages

Across the 13 *Salmonella* serotypes analyzed, cleavage patterns for the endonucleases that contain CTAG in the cleavage sites were drastically different and the sites at different genomic locations also had different levels of conservation; here we take XbaI cleavage as an example to illustrate the levels of conservation of the CTAG-containing sequences at different genomic locations. First of all, the XbaI cleavage sites within the tRNA encoding sequences had the highest level of conservation among the 13 *Salmonella* serotypes and even *E. coli* strain K12 as illustrated previously (Fig. 4 in [23]). Of great interest, XbaI 3 within an intergenic sequence (between STM1377–STM1378) is also conserved among the 13 *Salmonella* serotypes and *E. coli* strain K12; the potential biological function encoded by this genomic region is now under scrutiny. XbaI 4, 16 and 17, located in intergenic sequences between STM1622–STM1623, STM3405–STM3406 and STM3443–STM3444, respectively, are conserved in all analyzed *Salmonella* strains; characterization of these intergenic sequences for their potential roles in bacterial biology might provide novel insights into the evolution of bacteria. XbaI 26 in STM4362 (*hflX*) is conserved in all analyzed *Salmonella* strains, and XbaI 7, located in an intergenic sequence between STM2394–STM2394, is conserved in all *Salmonella* subgroup I strains analyzed here. Most other XbaI cleavage sites are specific either to one or a subset of *Salmonella* lineages (Supplementary Table S1). SpeI and other endonucleases having CTAG in the cleavage sites had similar general patterns as XbaI (data not shown). The distinct profiles of the CTAG-containing endonuclease cleavage sequences among the *Salmonella* serotypes make it possible to use these enzymes for delineating *Salmonella* into genetically well defined natural clusters, which would have to be further validated by comparisons between CTAG-containing cleavage site profiling and genome sequence information.

### Distinct CTAG-containing cleavage profiles to delineate *Salmonella* into natural lineages: correlation with core genome-based phylogenetics

**Figure 5:**
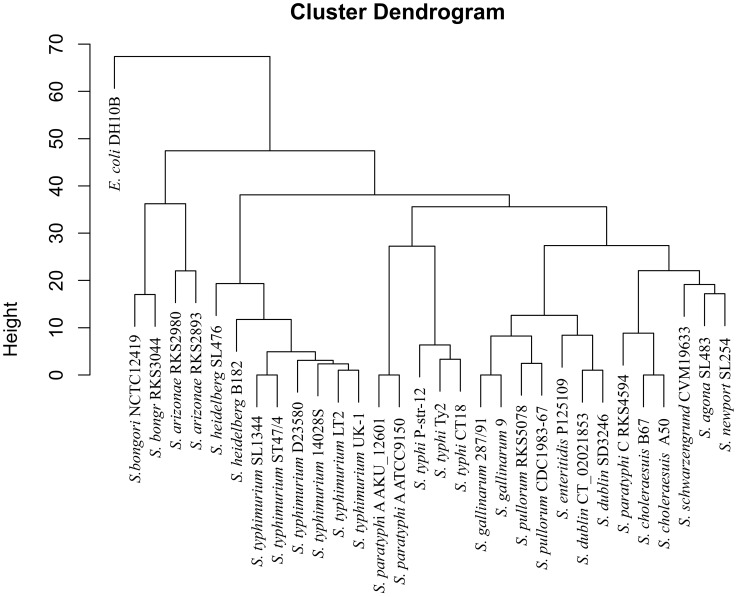
Phylogenetic tree constructed with the XbaI cleavage data based on numbers of conserved sites shared by subsets of the bacteria; B.

**Figure 6:**
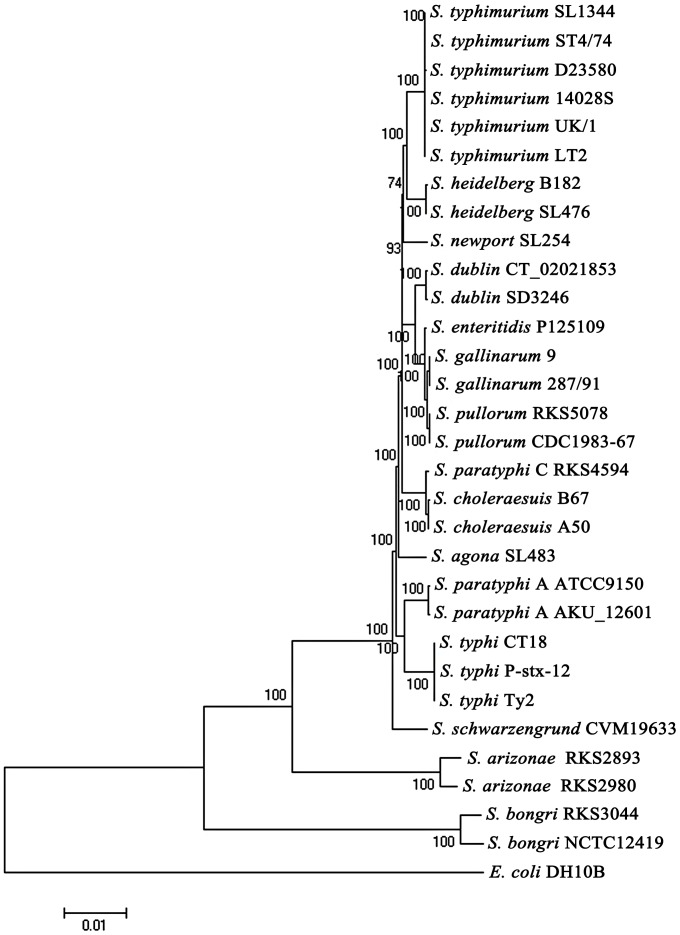
Phylogenetic tree constructed with concatenated core genome sequences, with the numbers beside the nodes indicating bootstrap values.

The high levels of conservation of the CTAG-containing cleavage sequences as exemplified by the distinct XbaI cleavage patterns in different *Salmonella* lineages suggest that profiling of such sequences may be used to delineate *Salmonella* into discrete natural lineages. To validate this, we conducted hierarchical clustering analysis on the XbaI cleavage profiling data among the *Salmonella* strains (Supplementary Table S2). Based on this analysis, we constructed a phylogenetic tree (Figure 5) and compared it to the core genome-based tree (Figure 6); the two trees revealed essentially the same phylogenetic relationships among the *Salmonella* strains.

## Discussion

In this study, we sampled a tiny portion of highly conserved sequences of the *Salmonella* genome, i.e., the CTAG-containing endonuclease cleavage sequences, as genomic signatures to probe the genetic uniqueness of individual *Salmonella* lineages and further test our hypothesis that bacteria dwell in nature as discrete genetic clusters. Findings from this may help evaluate and validate the genetic boundary concept, which is the core of our hypothesis. The highly similar genetic backgrounds in sharp contrast to the radical pathogenic differences among *Salmonella* make this genus of bacteria an ideal model for testing the hypothesis and for the studies of pathogenic evolution that turns benign organisms into infectious agents.

The topic on bacterial diversification, evolution and speciation has been a focus of extensive discussions, especially by investigators viewing from different angles and using different methods [24]–[32]. Originally, we initiated this work on the comparison between *S. typhimurium* and *S. typhi*, the former causing self-limiting gastroenteritis but the latter eliciting deadly typhoid fever in humans, to look for distinct genomic features that can be used to unambiguously divide them into discrete bacterial clusters, which, if demonstrated to exist, we call “natural species”, as they should be clusters of bacteria (“species”) formed by natural selection. We recently recognized and characterized clear-cut genomic divergence between them [33], which we defined as the genetic boundary. Such genetic boundaries have been documented in a broad range of bacteria, such as *Yesinia* and *Staphylococcus* [14]. In this study, we demonstrate that the selected subset of highly conserved sequences could reveal the genetic boundaries as clearly and reliably as whole genome analyses.

Compared to the whole genome strategies, CTAG-containing sequence profiling for *Salmonella* has several advantages. First, CTAG-containing cleavage sequence profiling by PFGE requires much less time and resources than genome sequencing strategies but still provides adequate information to delineate *Salmonella* into discrete genetic clusters, which is especially important when very large numbers of bacterial strains are involved; and second, the collection and analysis of CTAG-containing sequence data profiled by PFGE can be conducted in virtually any molecular biology laboratory equipped with the PFGE apparatus. Additionally, like whole genome sequences, the CTAG-containing cleavage sequence profiles are also objective and can be compared between laboratories and between platforms used. One case to be pointed out here is that monophyletic *Salmonella* serotypes like *S. gallinarum* may have diverse PFGE patterns (Fig. 1) of cleavage by XbaI or other endonucleases that have CTAG-containing cleavage sites, which may reduce the value of CTAG-containing endonuclease cleavage sequence profiling. However, even in such cases, well over 50% of the cleavage bands on PFGE are similar among the wild type strains, so creating no ambiguity.

We chose profiling the CTAG-containing endonuclease cleavage sequences to probe the *Salmonella* genomes for their genetic distinction also because it is a very useful and efficient method for a broad range of studies. For example, in addition to delineating the bacteria into discrete genetic clusters (i.e., natural species), which is our primary objective of this study, the profiling has a particular advantage in tracking the evolutionary scenarios of the *Salmonella* lineages, because the CTAG-containing sequences, though highly conserved in *Salmonella*, have been in the process of being eliminated from the genome by the VSP repair mechanism [15]. Assuming that all remaining CTAG-containing sequences through natural selection should be very important, we anticipated to see the gradual degeneracy processes of the CTAG-containing sequences among *Salmonella* as a whole. Specifically, the levels of conservation of the CTAG-containing sequences can be stratified by comparing their presence and degeneracy status (substitution of any of the CTAG nucleotides by transition or transversion) among the *Salmonella* lineages. For example, five XbaI cleavage sites are conserved not only across all *Salmonella* lineages compared in this study but also in *E. coli* (Supplementary Table S1). Other XbaI cleavage sites are either conserved among the *Salmonella* lineages but not in *E. coli*, or among *Salmonella* subgroup I lineages but not in other subgroups, or among strains of the same lineage, or specific to only particular strains of even the same lineage (in such cases, they are mostly in prophages or genomic islands). The differential profiles of the CTAG-containing cleavage sequences make each of the *Salmonella* lineages unique for identification, and the different patterns of sequence degeneracy among the *Salmonella* lineages (Supplementary Table S2) may provide important clues for their strategies in adapting to different environments (e.g., different host species).

Based on our results, we speculate the following evolutionary scenario that makes a small subset of highly conserved sequences to remain as a reliable and informative genetic signature of individual lineages. During the long process of CTAG elimination [15], each *Salmonella* lineage (dwelling in its own gene pool, [32]) accumulates nucleotide substitutions independently, leading to gradual degeneracy of the CTAG sequences in a particular way specific to each of the *Salmonella* lineages. Detailed analysis of the substituting and substituted nucleotides during the process of CTAG sequence degeneracy should provide novel insights into the strategy and mechanisms during the adaptation process of individual *Salmonella* pathogens, especially regarding their interaction with the host that they infect. We conclude that CTAG-containing sequence profiling can be used to unambiguously and efficiently delineate *Salmonella* into distinct genetic lineages, which are equivalent to the natural species of bacteria.

## Materials and Methods

### Bacterial strains

Bacterial strains used in this study along with the accession numbers of the sequenced genomes, are listed in Table 2; more detailed information on these bacteria can be found at the *Salmonella* Genetic Stock Center (http://www.ucalgary.ca/~kesander/). Bacteria were grown overnight at 37°C with shaking in Luria-Bertani (LB) broth or on LB plates. Stock cultures were stored at −70°C in LB broth with 25% glycerol.

**Table 2:** Bacterial strains used in this study^a^.

Strain	Accession number^b^	Reference
*S. typhimurium* LT2	AE006468	[23]
*S. typhimurium* 14028S	CP001363	
*S. typhimurium* SL1344	FQ312003	
*S. typhimurium* D23580	FN424405	
*S. typhimurium* ST4/74	CP002487	
*S. typhimurium* UK/1	CP002614	
*S. typhi* Ty2	AE014613	[17]
*S. typhi* CT18	NC_003198	
*S. typhi* P-stx-12	NC_016832	
*S. paratyphi* A ATCC9150	CP000026	[18]
*S. paratyphi* A AKU_12601	FM200053	
*S. paratyphi* C RKS4594	CP000857	[12], [36]
*S. agona* SL483	CP001138	
*S. dublin* CT_02021853	CP001144	
*S. dublin* SD3246	CM001151	
*S. enteritidis* P125109	AM933172	
*S. pullorum* RKS5078	CP003047	[21], [37]
*S. gallinarum* 287/91	AM933173	[22]
*S. gallinarum* SGSC2423	N/A	
*S. gallinarum* SGSC2292	N/A	
*S. gallinarum* SGSC2293	N/A	
*S. gallinarum* R1481	N/A	
*S. gallinarum* R1482	N/A	
*S. gallinarum* R1483	N/A	
*S. gallinarum* SARB21	N/A	
*S. choleraesuis* A50	CM001062	
*S. choleraesuis* SC-B67	AE017220	
*S. heidelberg* B182	NC_017623	
*S. heidelberg* SL476	CP001120	
*S. newport* SL254	CP001113	
*S. schwarzengrund* CVM19633	CP001127	
*S. arizonae* RKS2980	CP000880	
*S. arizonae* RKS2893	CP006693	
*S. bongori* NCTC 12419	FR877557	
*S. bongori* RKS3044	CP006692	

a See more detailed information on these bacterial strains at www.ucalgary.ca/~kesander.

b N/A means that the bacterial strain is not sequenced and the genome sequence was not needed for this study.

### Reagents and PFGE analyses of genomic DNA

I-CeuI, XbaI and AvrII were purchased from New England Biolabs, and proteinase K was from Roche. Most other reagents were from Sigma. Bacterial genomic DNA isolation, endonuclease cleavage with I-CeuI, XbaI and AvrII, and separation of the cleavage fragments were described previously [8], [17], [34]. Briefly, PFGE was used to separate DNA fragments cleaved by the endonucleases, and I-CeuI partial cleavage was used to lay out the overall genome structure of bacteria. PFGE was done in a CHEF DR II electrophoresis system (BioRad) at 5.6 V/cm with 0.5×TBE buffer as the running buffer.

### Genomic and statistics analysis tools

We determined the phylogenetic relationships of the bacteria based on their differences in the numbers of conserved CTAG-containing endonuclease cleavage sites common to subsets of *Salmonella* strains or sequence identity of genes common to them using the neighbor-joining (NJ) method, and the tree construction was done with MEGA4.0.2 [35] and CLUSTALW. The statistical analyses were performed by using software SPSS v20.

## Supporting Information

Table S1 **Profiles of XbaI cleavage sites in representative** ***Salmonella*** **genomes.**
(XLS)

Table S2 **Numbers of XbaI cleavage sites common to pairs of the** ***Salmonella*** **genomes.**
(XLS)
